# Biocompatibility and intradiscal application of a thermoreversible celecoxib-loaded poly-N-isopropylacrylamide MgFe-layered double hydroxide hydrogel in a canine model

**DOI:** 10.1186/s13075-015-0727-x

**Published:** 2015-08-20

**Authors:** Nicole Willems, Hsiao-yin Yang, Marloes L. P. Langelaan, Anna R. Tellegen, Guy C. M. Grinwis, Hendrik-Jan C. Kranenburg, Frank M. Riemers, Saskia G. M. Plomp, Eric G. M. Craenmehr, Wouter J. A. Dhert, Nicole E. Papen-Botterhuis, Björn P. Meij, Laura B. Creemers, Marianna A. Tryfonidou

**Affiliations:** Department of Clinical Sciences of Companion Animals, Faculty of Veterinary Medicine, Utrecht University, Yalelaan 108, Utrecht, 3584 CM The Netherlands; Department of Orthopedics, University Medical Center, Heidelberglaan 100, Utrecht, 3584 CX The Netherlands; Department of Materials Technology, TNO, De Rondom 1, Eindhoven, 5612 AP The Netherlands; Department of Pathobiology, Faculty of Veterinary Medicine, Utrecht University, Yalelaan 1, Utrecht, 3508 TD The Netherlands

## Abstract

**Introduction:**

Chronic low back pain due to intervertebral disc (IVD) degeneration is associated with increased levels of inflammatory mediators. Current medical treatment consists of oral anti-inflammatory drugs to alleviate pain. In this study, the efficacy and safety of a novel thermoreversible poly-N-isopropylacrylamide MgFe-layered double hydroxide (pNIPAAM MgFe-LDH) hydrogel was evaluated for intradiscal controlled delivery of the selective cyclooxygenase (COX) 2 inhibitor and anti-inflammatory drug celecoxib (CXB).

**Methods:**

Degradation, release behavior, and the ability of a CXB-loaded pNIPAAM MgFe-LDH hydrogel to suppress prostaglandin E_2_ (PGE_2_) levels in a controlled manner in the presence of a proinflammatory stimulus (TNF-α) were evaluated in vitro. Biocompatibility was evaluated histologically after subcutaneous injection in mice. Safety of intradiscal application of the loaded and unloaded hydrogels was studied in a canine model of spontaneous mild IVD degeneration by histological, biomolecular, and biochemical evaluation. After the hydrogel was shown to be biocompatible and safe, an in vivo dose–response study was performed in order to determine safety and efficacy of the pNIPAAM MgFe-LDH hydrogel for intradiscal controlled delivery of CXB.

**Results:**

CXB release correlated to hydrogel degradation in vitro. Furthermore, controlled release from CXB-loaded hydrogels was demonstrated to suppress PGE_2_ levels in the presence of TNF-α. The hydrogel was shown to exhibit a good biocompatibility upon subcutaneous injection in mice. Upon intradiscal injection in a canine model, the hydrogel exhibited excellent biocompatibility based on histological evaluation of the treated IVDs. Gene expression and biochemical analyses supported the finding that no substantial negative effects of the hydrogel were observed. Safety of application was further confirmed by the absence of clinical symptoms, IVD herniation or progression of degeneration. Controlled release of CXB resulted in a nonsignificant maximal inhibition (approximately 35 %) of PGE_2_ levels in the mildly degenerated canine IVDs.

**Conclusions:**

In conclusion, this study showed biocompatibility and safe intradiscal application of an MgFe LDH-pNIPAAM hydrogel. Controlled release of CXB resulted in only limited inhibition of PGE_2_ in this model with mild IVD degeneration, and further studies should concentrate on application of controlled release from this type of hydrogel in animal models with more severe IVD degeneration.

**Electronic supplementary material:**

The online version of this article (doi:10.1186/s13075-015-0727-x) contains supplementary material, which is available to authorized users.

## Introduction

Chronic low back pain is a debilitating disorder associated with intervertebral disc (IVD) degeneration [1]. As the exact pathogenesis is still poorly understood, current surgical and medical treatments aim at alleviating symptoms. Inhibiting or reversing the degenerative process by using advanced methods like cell and tissue engineering are in development, but are not clinically applicable thus far. In degenerative disc diseases, the specific composition of the nucleus pulposus (NP) and annulus fibrosus (AF) is disturbed, since the delicate equilibrium shifts toward the catabolic pathways [2, 3]. In the NP this results in a change from an extracellular matrix (ECM) rich in proteoglycans and type II collagen, to a tissue containing mainly type I collagen, and in the AF in a loss of lamellar organization [4, 5]. The loss of proteoglycans causes a decrease in the water-binding capacity of the NP and together with the changes in the AF, compromises the structural functionality of the IVD [5].

A variety of inflammatory mediators has been investigated for their role in the catabolic processes of IVD degeneration; targeting the inflammation process is one of the emerging treatment strategies of chronic low back pain and IVD degeneration. Herniated degenerative disc tissue has been shown to spontaneously produce increased amounts of matrix metalloproteinases (MMPs), nitric oxide, prostaglandin E_2_ (PGE_2_) and interleukin 6 (IL-6), and to express interleukin 1 (IL-1), interleukin 8 (IL-8), tumor necrosis factor alpha (TNF-α) [6, 7]. TNF-α and IL-1 upregulate expression of matrix-degrading enzymes by NP cells [4, 8, 9]. Furthermore, elevated levels of IL-1 and PGE_2_ have been associated with aging and degeneration of the IVD [9, 10]. In the NP, PGE_2_ negatively affects matrix integrity by inhibiting proteoglycan synthesis, possibly mediated by a decrease in insulin growth factor 1 and an increase in matrix-degrading enzymes [10].

PGE_2_ is a well-known prostanoid and plays an important regulatory role in physiological as well as pathological processes like intervertebral disc degeneration. It is synthesized by two cyclooxygenase (COX) isoforms, COX-1 and COX-2, by conversion of arachidonic acid into prostaglandin H_2_ (PGH_2_) and isomerization of PGH_2_ to PGE_2_ by prostaglandin E synthases (PTGES). COX-1 is constitutively expressed in most tissues and is associated with the production of baseline PGE_2_ levels important for homeostasis. In contrast, COX-2 expression is highly restricted under physiological conditions, but can be rapidly induced in response to inflammatory stimuli and is therefore believed to play an important role in the PGE_2_ production involved in degenerative processes [11, 12]. Selective COX-2 inhibitors have been developed to reduce PGE_2_ production via this pathway. In several clinical trials the efficacy of COX-2 inhibitors in patients with low back pain has been established [13, 14]. However, their widespread application is hampered by severe side effects, such as cardiotoxicity. Although these inhibitors can be effectively introduced into the avascular IVD by intradiscal injection, they would achieve only short-lived clinical effects. Delivering drugs by using controlled release systems, e.g., hydrogels, would be a more attractive alternative to bolus injections, as a higher loading dose and long-term delivery can be accomplished by a minimum of intradiscal interventions [15, 16].

Temperature-sensitive poly-N-isopropylacrylamide (pNIPAAM)-based hydrogels have been extensively used in the field of controlled release [17]. These gels could be particularly suitable for intradiscal injection as they are liquid at room temperature, and hence injectable through small-diameter needles and form a solid gel at 37 °C, preventing leakage of injected materials from the IVD [18]. In this study a hybrid thermoreversible biodegradable hydrogel served as a controlled release platform for the specific COX-2 inhibitor celecoxib (CXB). This release system consists of lower critical solution temperature (LCST) polymers with a low molecular weight, based on pNIPAAM with a sulfonate end group, ionically linked to a network of biodegradable platelet-type MgFe layered double hydroxide (LDH) nanoparticles. LDH particles possess a positive surface charge, which can interact with the negatively charged LCST polymers. At room temperature these hybrid structures are simply made by mixing the polymer with the MgFe-LDHs and subsequent dispersion in water. This results in a solution of low viscosity that can easily be injected into the NP via a 29G needle. At body temperature (37 °C), physical entanglements are formed due to hydrophobic interactions between the LCST polymers, resulting in the formation of a hybrid network, as polymers are linked to the MgFe-LDH particles. Furthermore, the easily ionizable carboxylic groups of CXB can interact with the biodegradable LDHs, which makes this unique for drug release. We hypothesize that pNIPAAM MgFe-LDH hydrogels are suitable vehicles for delivering a COX-2 inhibitor into the IVD, to reduce intradiscal PGE_2_ levels over time in a dog model with spontaneous IVD degeneration, showing pathophysiological aspects similar to those in human [19, 20].

## Materials and methods

After synthesis, preparation, and rheological analysis of the hydrogel, the degradation and release behavior of the CXB-loaded hydrogel was evaluated. Furthermore, in an in vitro model in the presence of a proinflammatory stimulus (TNF-α) the ability of the CXB-loaded hydrogels to suppress PGE_2_ levels in a sustained manner was evaluated. Thereafter, biocompatibility upon subcutaneous injection was studied in mice. Safety of intradiscal application of the loaded and unloaded hydrogel was studied in a canine model of spontaneous mild IVD degeneration. After the hydrogel was shown to be biocompatible and safe, a follow-up dose–response in vivo study was performed in order to determine safety and efficacy of the pNIPAAM MgFe-LDH hydrogel for intradiscal controlled delivery of CXB.

### Synthesis and preparation of pNIPAAM MgFe-LDH hydrogels

The poly-N-isopropylacrylamide (pNIPAAM) polymer with sulfonate end group was synthesized as reported previously [21] and the modified synthesis is described in detail in Additional file 1. To formulate the hydrogel, the pNIPAAM polymer was added to the LDH suspension in a vial and subsequently placed on a tube roller mixer for 48 h at room temperature and sterilized by gamma radiation (25 kGy, Isotron Nederland BV, Ede, The Netherlands). The final hydrogel contained 16 wt % pNIPAAM, 3.3 wt % MgFe LDH and water.

### Rheological analysis of the pNIPAAM MgFe-LDH hydrogels

The viscoelastic properties of the unloaded pNIPAAm MgFe-LDHs were determined by using an Anton Paar MCR301 rheometer (Anton Paar Ltd., St. Albans, UK) with an oscillatory parallel plate geometry (50 mm diameter) with a constant strain of γ = 0.5 % at a frequency of f = 1 Hz. Temperature was increased from 22 °C to 37 °C at a rate of 15 °C/min. This heating rate was chosen based on calculating the minimum rate of heat transfer based on estimating the energy needed to heat up the hydrogel, by taking into account the surface of the hydrogel and the minimum temperature difference between the LCST and body temperature. LCST is the critical temperature above which the hydrogel undergoes a phase transition from a soluble to an insoluble state. This estimation is described in detail in Additional file 1. The gelling was recorded by measuring the complex shear modulus |G*|, which is a common parameter to determine the strength of a viscoelastic material like a hydrogel. The complex shear modulus |G*| is correlated to the storage modulus (G’) and loss modulus (G”). The storage modulus is a measure of the deformation energy stored in the sample during the shear process (elastic behavior), whereas the loss modulus is a measure of the energy dissipated in the sample during the shear process (viscous behavior), and is lost to the sample afterward (viscous behavior). The relation between these parameters is the following:

$$ \left|G*\right|=\sqrt{{\left(G\hbox{'}\right)}^2+{\left(G"\right)}^2} $$

**Fig. 1:**
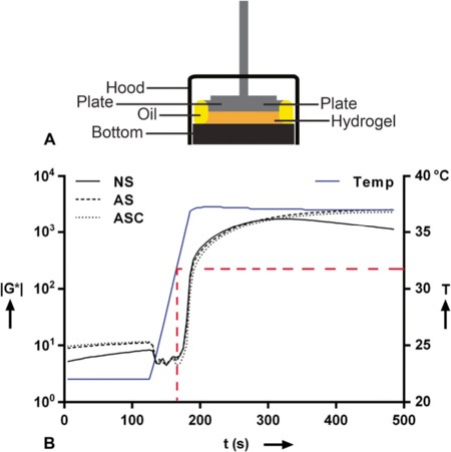
Rheological setup and results of the complex shear modulus |G*| of the pNIPAAM MgFe-LDH hydrogel. **a** Configuration of the rheological setup to measure |G*| of the pNIPAAM MgFe-LDH hydrogel. **b** |G*| of the pNIPAAM MgFe-LDH hydrogel as a function of temperature. The *dashed red line* connects the time point where the |G*| starts to increase to the temperature curve, and indicates a lower critical solution temperature (LCST) of 32 °C. *AS* after sterilization*, ASC* after sterilization separate components, *NS* nonsterilized. G* complex shear modulus, *LDH* layered double hydroxide, *pNIPAAM* poly-N-isopropylacrylamide

Hydrogel samples were placed on the lower plate, and the upper plate was lowered to a 0.5 mm gap. The configuration of the rheological setup is shown in Fig. 1a. The viscoelastic properties of the loaded hydrogel were not determined. The CXB concentrations were as low as 10^−6^ M to 10^−4^ M, i.e., 0.38–38 mg of celecoxib per liter, or 0.0038–3.8 × 10^-5^ wt %, and were not influencing the rheological properties.

### Degradation and release behavior of CXB-loaded pNIPAAM MgFe-LDH hydrogels

In vitro, the controlled release of CXB from hydrogels was measured in phosphate-buffered saline (PBS) (pH 7.4, 44 mM Na_2_HPO_4_, 9 mM NaH_2_PO_4_, 72 mM NaCl, 0.02 % wt NaN_3_) and 0.2 % Tween 80® (polyoxyethylenesorbitan monooleate; Sigma-Aldrich Chemie B.V., Zwijndrecht, The Netherlands). Tween 80® was added to the buffer in order to increase the solubility of CXB [22] and thereby simulate the in vivo situation. CXB-loaded pNIPAAM MgFe-LDH suspension was prepared by adding 6 or 10 mg/ml of CXB to the dispersion and stirring with a stirring bar for 2 days. A volume of 1 ml of CXB-loaded pNIPAAM MgFe-LDH suspension was pipetted into a vial and placed for 30 min at 37 °C to ensure gelation of the hydrogel, and afterward covered with 14 ml warm (37 °C) PBS/Tween 80® solution. The release experiment was performed at 37 °C. At day 1, 2, 5, 8, 15, 22, and 31, 12 ml of the buffer solution was removed in order to analyze CXB and Mg concentrations and 12 ml of fresh buffer was added. CXB concentrations were determined in a volume of 100 μl by using ultra-performance liquid chromatography (UPLC) as described in detail recently by Petit *et al.* [23]. In vitro degradation was determined by measuring the relative cumulative release of Mg into the medium. Mg concentrations were determined by using a Prodigy High Dispersion Inductively Coupled Optical Emission Spectometry (ICP-OES) system (Leeman Labs, St Charles, IL, USA). Standards were prepared by using multi-element (23 elements in diluted nitric acid) standard solution IV (1000 mg/l) (Merck Millipore, Darmstadt, Germany). A volume of 0.5 ml of the solutions from the degradation experiment was diluted in 100 ml aqueous 1N HNO_3_, and subsequently diluted again tenfold in 1N HNO_3_. The effects of CXB loading (10 mg/ml versus 6 mg/ml), LDH content (single versus double) and type of LDH (Mg_3_Fe versus Mg_2.5_Fe) of the gels on release behavior and in vitro degradation were also investigated, as well as CXB solubility effects by using a buffer containing PBS and 0.2 % or 2 % Tween 80®.

### Controlled release of CXB in vitro

**Fig. 2:**
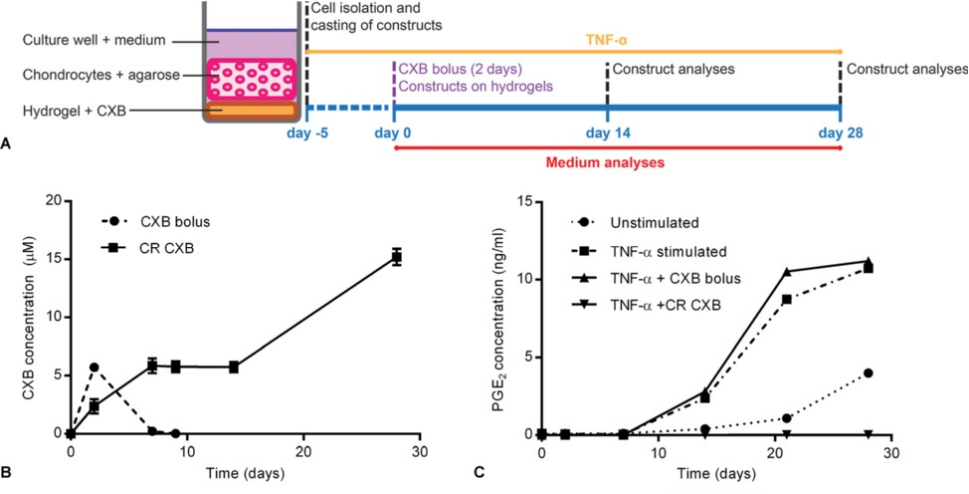
In vitro controlled release of CXB results into sustained suppression of PGE_2_ levels in the presence of a proinflammatory stimulus. **a** Experimental setup to evaluate the controlled release of celecoxib (CXB) in vitro. During the first 5 days of culturing, three-dimensional chondrocyte constructs were stimulated with a proinflammatory cytokine TNF-α (10 ng/ml). At day 0 of the experiment, a 10 μM bolus injection of CXB was applied for 2 consecutive days or pNIPAAM MgFe-LDH hydrogels loaded with 1 mg/ml CXB (CR CXB). Media were refreshed every 2–3 days and collected on days 0, 2, 7, 9, 11, 14, 21, and 28. On days 14 and 28 the constructs were evaluated for cell viability. **b** Celecoxib (CXB) concentrations (μM) were measured in medium samples after administering the bolus injection of CXB or the pNIPAAM MgFe-LDH hydrogel loaded with 1 mg/ml CXB (CR CXB) **c**. PGE_2_ concentrations (ng/ml) measured in medium samples in the following conditions: unstimulated, stimulated with 10 ng/ml TNF-α (TNF-α stimulated), TNF-α stimulated in the presence of a 10 μM bolus injection of celecoxib (CXB bolus) for 2 consecutive days, or 1 mg/ml CXB-loaded pNIPAAM MgFe-LDH hydrogels (CR + CXB). Data are expressed as mean ± standard deviation. *CR* control, *LDH* layered double hydroxide, *PGE*_*2*,_ prostaglandin E_2_, *pNIPAAM* poly-N-isopropylacrylamide, *TNF-α* tumor necrosis factor alpha

The balance between anabolic and catabolic pathways in articular chondrocytes as well as NP cells can be directed toward catabolism by TNF-α [24, 25]. Bovine articular chondrocytes were used in the in vitro experiments, as they were more easily available in our laboratory. Articular chondrocytes were isolated from bovine carpometacarpal joints by enzymatic digestion overnight with 2 mg/ml collagenase A (Roche Diagnostics Deutschland GmbH, Mannheim, Germany) at 37 °C. Chondrocytes were seeded at 5 × 10^6^ cells/ml density (P0) into cylindrical (diameter and height 6 mm) 2 % agarose (Type VII, Sigma-Aldrich Chemie B.V.) constructs and left to gel at room temperature. The constructs were then cultured in 12-well plates in high-glucose Dulbecco’s modified Eagle’s medium (hgDMEM; Gibco, Life Technologies Europe, Bleiswijk, The Netherlands) with 20 % fetal bovine serum (Greiner Bio-One, Alphen aan Den Rijn, The Netherlands), 0.1 % amphotericin (Sigma-Aldrich Chemie B.V.), 1 % Pen-Strep (Biochrom GmbH, Berlin, Germany), 1 % nonessential amino acids (Lonza, Basel, Switzerland) 1 % essential amino acids (Lonza), and 50 mg/ml ascorbate 2-phosphate (Biochrom). During the first 5 days of culturing, these constructs were stimulated with 10 ng/ml TNF-α to induce an inflammatory response reflected by elevated PGE_2_ levels (Fig. 2a). A concentration of 1 μM CXB has been described to effectively lower PGE_2_ levels in osteoarthritic chondrocytes and corresponds with mean pharmacological plasma levels [26]. CXB was dispersed in the pNIPAAM MgFe-LDH mixture at a concentration of 0.1 mg/ml, aiming to establish a concentration of approximately 1 μM CXB per culture medium renewal over the 28-day culture period. Controlled release of the CXB is achieved by dissolution of the CXB crystals present a depot within the hydrogel and diffusion of the solubilized CXB. A volume of 100 μl of the hydrogel suspension was pipetted at the bottom of a 12-well plate and placed in an incubator at 37 °C to ensure gelation of the hydrogels. Subsequently, defined as day 0 of the experiment, cell constructs and culture medium were added to the 12-well plate. For the “bolus injection” of CXB, only cell constructs were placed on the bottom of the well, and CXB was added to the medium every 2 days, starting at day 0, at a concentration of 1 μM. Media were renewed every 2–3 days, collected on days 0, 2, 7, 9, 11, 14, 21, and 28 and stored at −80 °C for analysis of CXB content. For the in vivo experiments based on formulation of higher doses of CXB, in vitro experiments were also carried out with a higher dosage of CXB to evaluate release profiles at higher dosing. To this end, 1 mg of CXB-loaded per ml of hydrogel, aiming to establish a concentration of 10 μM CXB per culture medium renewal was used. Conditioned media were analyzed for CXB content and PGE_2_ levels. Inhibition of COX-2 activity was determined by measuring PGE_2_ in culture medium. A colorimetric competitive enzyme immunoassay kit (PGE_2_ EIA kit, Enzo Life Sciences BVBA, Antwerp, Belgium) was used to determine PGE_2_ levels in culture medium according to the manufacturer’s instructions.

### In vivo biocompatibility in mice after subcutaneous implantation

All animal procedures were approved and performed in accordance with the guidelines set by the Animal Experiments Committee (DEC) of Utrecht University (experiment numbers: DEC 2010.III.03.046; DEC 2012.III.05.046; and DEC 2013.III.02.017). Six healthy female adult (8–10 weeks old) BALB/c mice (Harlan-Olac Ltd., Bicester, UK) were used for testing biocompatibility and biosafety of the pNIPAAM MgFe-LDH polymer hydrogel and seven other biomaterials. Four different biomaterials were injected at least 1 cm apart from each other into the dorsal subcutaneous tissue of each mouse in a randomized fashion. Buprenorphine 100 μg/kg was given intraperitoneally (i.p.) as premedication and analgesic and subsequently all animals were anesthetized with isoflurane via an induction mask (vaporizer setting 2.5 %) in a 1:1 oxygen:air mixture. A blood sample was drawn to perform a white blood cell count and differentiation at day 0, to rule out systemic inflammation. A volume of 200 μl of each biomaterial was injected subcutaneously with a 27G needle under sterile conditions. PBS (200 μl) served as a control. All injection sites were marked with a waterproof marker. Immediately after injection, Dermabond® (Ethicon, Cornelia, GA, USA) was applied to the injection site to prevent leakage and the injection site was heated by an infrared lamp for 1 min. Mice were monitored daily for signs of distress or pain (e.g., lethargy, weight loss, automutilation, and abnormal posture) and injection sites were monitored for inflammation (e.g., swelling, redness, pain, and heat). Three animals were sacrificed 7 days after injection, and three after 28 days. At the end of the experimental period, animals were anesthetized with isoflurane, blood was collected by cardiac puncture for white blood cell count and differentiation, and euthanasia was performed by cervical dislocation. The injection sites were removed for histological analysis. Tissues were fixated in a 4 % neutral-buffered formaldehyde solution (Klinipath B.V., Duiven, The Netherlands) and after fixation routinely embedded in paraffin. Sections of 4 μm were stained with hematoxylin and eosin. Infiltration of inflammatory cells, giant cells, necrosis, neovascularization, fatty infiltration, and the encapsulation of the biomaterial by a fibrotic capsule were histologically assessed as parameters for a biological response at the application site, at 7 and 28 days by a blinded board-certified veterinary pathologist (GG) and the principal investigator (NW) using an Olympus BX41 microscope (Olympus Europa GmbH, Hamburg, Germany).

### Intradiscal application of CXB-loaded pNIPAAM MgFe-LDH hydrogels in laboratory beagle dogs

Data from two in vivo studies in beagle dogs were combined and analyzed. Both studies were set up as randomized block designs. In the first study CXB-loaded (7.7 μM) and unloaded pNIPAAM MgFe-LDH hydrogels, a bolus injection of CXB (7.7 μM) and 0.9 % NaCl were intradiscally injected. In other levels two other materials irrelevant to this study were injected. The second study served as a dose–response study, including a 10- and 100-fold higher dosage of CXB (77 μM and 770 μM) in addition to the 7.7 μM dose. For preparation of the CXB-loaded hydrogels, CXB was prepared from a CXB stock solution in ethanol (60 μg/ml) by sterile filtration. Water was added to this ethanolic solution of CXB to obtain a dispersion with small CXB crystals (diameter approximately 1 μm). This dispersion was freeze-dried overnight and the pNIPAAM MgFe-LDH mixture was added and incubated overnight on a tube roller mixer at room temperature.

In total 18 intact female beagle dogs (Harlan, Gannat, France) with a median age of 1.7 years (range 1.3–1.8 years) and a median weight of 8.4 kg (range 6.2–13.8 kg) were used. Nine dogs with a median age of 1.6 years (range 1.3–1.8 years) and a median weight of 8.2 kg (range 6.2–11 kg) were used in the first study. Nine dogs with a median age of 1.7 years (range 1.6–1.8 years) and a median weight of 9.3 kg (range 8.3–13.8 kg) were used in the second study. All dogs underwent general, orthopedic, and neurologic examination by a board-certified veterinary surgeon (BM).

### Surgical procedure

To determine the grade of degeneration of the IVDs prior to surgery, magnetic resonance (MR) images of the lumbar vertebral column were obtained in fully anesthetized dogs. A blood sample was drawn from the jugular vein to assess white blood cell count and differentiation, to exclude systemic inflammation. Dogs were placed in a dorsal recumbent position and throughout the complete scan protocol heart rate, respiration rate, temperature, carbon dioxide, and oxygen levels were monitored. The MR imaging was performed using a 0.2 Tesla open magnet (Magnetom Open Viva, Siemens AG, Munich, Germany). All lumbar IVDs were assessed according to the Pfirrmann score by a veterinary radiologist on sagittal T2-weighted fast spin echo (FSE) images (3.0 mm slices, repetition time (TR) 4455 ms, echo time (TE) 117 ms) [27]. Only lumbar IVDs with a Pfirrmann score II were included for injection.

The anesthesia protocol during surgery was similar to the one used during MR scanning. Analgesia was provided by a combination of fentanyl (loading dose 10 μg/kg, 15–20 μg/kg/h continuous rate infusion, c.r.i.) and ketamine (0.5 mg/kg loading dose, 10 μg/kg/min c.r.i.) intravenous (i.v.). Throughout the complete procedure heart rate, respiration, temperature, carbon dioxide, oxygen levels, and blood pressure (noninvasive) were monitored. Surgical sites were prepared according to standard protocol. A detailed description of the surgical procedure has been described previously [28]. Briefly, dogs were positioned in a right recumbent position to expose and inject the T13-L1 until L6-L7 via a left lateral approach. To diminish injury of the iliopsoas muscle and sciatic nerve traction injury, the surgical approach in the second study was adjusted and L6-L7 and L7-S1 were injected via a dorsal approach, while the dogs were positioned in ventral recumbency. In the first study a 100 μl syringe (7638–01 Model 710 RN, Hamilton Company USA, Reno, NV, USA), and in the second study a 100 μl gastight syringe (7656–01 Model 1710 RN) with a 29G needle (25 mm, 12° beveled point; Hamilton Company USA) was used to inject 30 μl of the earlier mentioned compounds through the AF into the NP. The smallest possible needle diameter was chosen to minimize injury to the treated IVDs. Wound closure was performed according to standard protocol. Postoperative pain management in all dogs consisted of methadone 0.3 mg/kg intramuscular (i.m.) quaque (q).6.h. during the first 24 h postoperatively and buprenorphine 20 μg/kg i.m. q.4.h. and/or tramadol 2–5 mg/kg per os (p.o.) q.6.h. the following 7 days. All dogs were treated postoperatively with antibiotics (amoxicillin/clavulanic acid 12.5 mg/kg q.12.h p.o.) during 5 days. Dogs were monitored daily throughout the study by a veterinarian to assess pain symptoms according to the short form of the Glasgow composite pain scale. Dogs that showed signs of pain, received tramadol and/or buprenorphine and/or gabapentin (5 mg/kg p.o. q.12.h). Furthermore, animals were monitored daily by a veterinarian for clinical signs of illness, neurologic deficits and lameness.

### Injected substances

In the first study spontaneously degenerated IVDs (Pfirmann grade 2) of the dogs were injected with a volume of 30 μl of NaCl 0.9 % (sham), a bolus of CXB (7.7 μM), a CXB-loaded (7.7 μM) and an unloaded pNIPAAM MgFe-LDH hydrogel. Based on studies in cadaveric spines (unpublished data, N. Willems, and B.P. Meij) a volume of 30 μl could be injected into the NP without substantial resistance. The volume of 30 μl contained 7.7 μM × 10^−6^ M CXB, to achieve a final concentration of 1 μM (=7.7 × 10^−6^ × (30 μl gel/230 μl NP volume plus gel)) for the bolus of CXB, in the canine NP of beagle laboratory dogs with a mean weight of 8–9 kg and taking into account the volume of the nucleus (200 μl) [29]. All substances were injected into the IVDs in the T12-L6 spinal segment in a randomized fashion, except for the sham treatment (NaCl 0.9 %), which was injected into T12-T13. An interim statistical analysis was performed after the first study to evaluate treatments and study design. Results were used to perform a new power analysis and to adapt the study design of the second study. In the second study, all substances were administered in a random order within each animal and IVDs of the T12-S1 spinal segment were injected with NaCl 0.9 %, a bolus of CXB (7.7 μM), CXB-loaded (7.7 μM, 77 μM and 770 μM) hydrogels, and an unloaded pNIPAAM MgFe-LDH hydrogel. IVDs adjacent to those injected with hydrogel loaded with the highest dose of CXB (770 μM) remained untreated.

### Postmortem collection of materials

Dogs were euthanized 4 weeks postinjection. First, they were sedated with dexmedetomidine 0.04 mg/kg i.v., followed by pentobarbital 200 mg/kg i.v. Immediately after euthanasia, the vertebral column (T12-S1) was harvested by using an electric multipurpose saw (Bosch, Stuttgart, Germany). All muscles were removed and the vertebrae were transected transversely with a band saw (EXAKT tape saw, EXAKT Advanced Technologies GmbH, Norderstedt, Germany), resulting in nine spinal units (endplate–IVD–endplate). These units were then transected sagittally by using a diamond band pathology saw (EXAKT 312 saw; EXAKT diamond cutting band 0.1 mm D64; EXAKT Advanced Technologies GmbH), generating two identical parts. One half was resected with a surgical knife by removing the endplate and the vertebra attached to it on one side, and the remaining IVD tissue was snap frozen in liquid nitrogen stored at −80 °C for biochemical and biomolecular analyses. The other half was photographed (Olympus VR-340, Olympus Europa GmbH) for macroscopic evaluation of the IVD (Thompson score, see below) and stored for 14 days in 50 ml of 4 % buffered formaldehyde at 4 °C for histological analyses.

### Histology, COX-2 immunohistochemistry, and TUNEL assay

Samples were decalcified in 35 % formic acid and 6.8 % sodium formate in a microwave oven (Milestone Microwave Laboratory Systems, Bergamo, Italy) overnight at 37 °C, for 7 nights [30] and embedded in paraffin. Five-μm-thick sections were stained with hematoxylin and eosin and with picrosirius red/alcian blue and evaluated according to a grading scheme according to Bergknut et al. [31]. Histological slides were scored blinded and in random order by two independent investigators (NW, AT) using an Olympus BX41 microscope (Olympus Europa GmbH). In case of doubt, samples were also scored by a board-certified veterinary pathologist (GG). All photographs of the macroscopy of the IVD segments were evaluated by two independent blinded investigators (NW, AT) according to the Thompson grading scheme, which has been validated in dogs [32].

Immunohistochemistry for COX-2 was performed on 5 μm sections mounted on KP-plus glass slides. After deparaffinization and rehydration, sections were treated with Dual Endogenous Enzyme Block (Dako S2003, Dako, Carpinteria, CA, USA) for 10 min at room temperature to block nonspecific endogenous peroxidase, followed by two washing steps each of 5 min with Tris-buffered saline with 1 % Tween 20® (TBST). Sections were treated with Tris-buffered saline (TBS) bovine serum albumin (BSA) 5 % solution to block nonspecific binding for 60 min at room temperature, were carefully rinsed and subsequently incubated with a primary mouse anti-human monoclonal COX-2 antibody (Cayman Chemical, Ann Arbor, MI, USA) diluted 1:50 in TBS-BSA 5 % overnight at 4 °C. The following day sections were incubated with peroxidase-labeled polymer (Envision™ anti-mouse K4001, Dako). Antibody binding was visualized by using diaminobenzidine (DAB; Dako). Sections were counterstained with hematoxylin solution (Hematoxylin QS, Vector Laboratories Ltd., Peterborough, UK) and mounted in permanent mounting medium.

A commercial available terminal deoxynucleotidyl transferase dUTP nick-end labeling (TUNEL; Merck Millipore, Darmstadt, Germany) assay was used according to the manufacturer’s instructions to determine apoptosis. The percentage of COX-2-positive and TUNEL-positive chondrocytes over the total number of cells was determined by manual counting in the NP, and in the ventral (VAF) and dorsal AF (DAF), by two blinded independent investigators (NW, SP).

### Biomolecular and biochemical analyses

Cryosections (60 μm) of the spinal units were cut with a cryostat (Leica CM1800 cryostat, Leica Microsystems Inc., Bannockburn, IL, USA) and collected on RNAse-free glass slides. The NP and AF tissues were separated and half of the slides were collected in respectively 400 μl and 750 μl Ambion® KDalert™ lysis buffer solution (Life Technologies) in the first study, and in Complete Lysis-M EDTA-free buffer (Roche Diagnostics Nederland B.V., Almere, The Netherlands) in the second study and stored at −80 °C until biochemical analyses were performed. The other half was collected in 300 μl RLT buffer containing 1 % β-mercaptoethanol (Qiagen, Venlo, The Netherlands) and stored at −80 °C until biomolecular analyses were performed.

Quantitative PCR (qPCR) was performed to assess the effects of (controlled release of) CXB at gene expression levels of the NP with regards to: 1) ECM anabolism: aggrecan (*ACAN*), collagen type II (*COL2A1*), collagen type I (*COL1A1*); 2) ECM catabolism: a disintegrin and metalloproteinase with thrombospondin motifs 5 (*ADAMTS5*), matrix metalloproteinase 13 (*MMP13*), tissue inhibitor of metalloproteinase 1 (*TIMP1*); 3) inflammation: tumor necrosis factor alpha (*TNFA*), interleukin-1β (*IL1B)*, interleukin-6 (*IL6*) and interleukin-10 (*IL10*); 4) COX pathway and PGE_2_ synthesis: prostaglandin E synthase 1 (*PTGES1*), prostaglandin E synthase 2 (*PTGES2*), cyclooxygenase 1 (*COX1*), and cyclooxygenase 2 (*COX2*); 5) notochordal markers: brachyury (*T*), cytokeratin-8 (*CK8*), cytokeratin-18 (*CK18*); 6) the indirect effect of CXB on Wnt signaling pathway: axin-2 (*AXIN2*), c-Myc (*c-Myc*) and cyclin-D1 (*CCND1*) and 7) apoptosis: caveolin-1 (*CAV1*), caspase 3 (*CASP3*), fas ligand (*FasL*) and Bcl-2 (*BCL2*). The primer pairs used for qPCR are given in Additional file 2.

The RNeasy Fibrous Tissue Mini Kit (Qiagen, Venlo, The Netherlands) was used to isolate total RNA. To maximize RNA yield, the incubation period with proteinase K was reduced to 5 min. After on-column DNase-I digestion (Qiagen RNase-free DNase kit) RNA was quantified by using a NanoDrop 1000 spectrophotometer (Isogen Life Science B.V., Ijsselstein, The Netherlands). cDNA was synthesized from 20 ng total RNA in a total volume of 15 μl using the iScript™ cDNA Synthesis Kit (Bio-Rad Laboratories B.V., Veenendaal, The Netherlands). qPCR was performed in duplicate using an iCycler CFX384 Touch™ thermal cycler, and IQ SYBRGreen Super mix (Bio-Rad Laboratories). All dog-specific primers were designed in-house using Perlprimer [33] except for *MMP13* [34]. Primer specificity was evaluated with BLAST, and the designed amplicon was tested for secondary structures using MFold [35]. Primers were purchased from Eurogentec, Maastricht, The Netherlands. Amplification efficiencies ranged from 80 to 115 %. Relative expression levels were determined by normalizing the cycle threshold (Ct) value of each target gene by the mean Ct value of three reference genes, i.e., glyceraldehyde 3-phosphate dehydrogenase (*GAPDH*), ribosomal protein S19 (*RPS19*), and TATA-binding protein (*TBP*).

To measure glycosaminoglycan (GAG) and DNA content in the NP and AF, samples in Ambion® KDalert™ lysis buffer solution were homogenized in a tube rotator O/N at 4 °C, whereas samples in Complete Lysis-M EDTA-free buffer were homogenized in a TissueLyser II (Qiagen) for 2 × 30 s at 20 Hz. The supernatant and pellet of each NP and AF were digested overnight in a papain buffer (250 μg/ml papain (Sigma-Aldrich) in 50 mM EDTA and 5 mM L-cysteine) at 60 °C. GAG content was quantified by using a 1,9-dimethylmethylene blue assay [36]. The Quant-iT™ dsDNA Broad-Range assay kit in combination with a Qubit™ fluorometer (Invitrogen, Carlsbad, CA, USA) was used in accordance with the manufacturer’s instructions to determine DNA content in the papain-digested NP and AF supernatant and pellets. DNA content in the supernatants of the NP and AF were negligible and therefore not included in the total content.

PGE_2_ levels were measured with the same colorimetric competitive enzyme immunoassay kit (PGE_2_ high sensitivity EIA kit, Enzo Life Sciences BVBA) that was used for the in vitro experiments. Both buffers that were used to lyse tissue were validated and standards were diluted in the same lysis buffer as the samples, which did not show strong interference with the performance of the kit. Total GAG content and PGE_2_ levels were normalized for DNA content in the pellet and were measured in the NP as well as the AF.

### Statistical analyses

Power analyses were performed prior to both in vivo studies by using free software [51], and are described in detail in Additional file 3. PGE_2_/DNA in the NP was considered to be the main read-out parameter. Biochemical and biomolecular data were analyzed by using the R statistical software, package 2.15.2. A linear mixed-effects model was used to analyze the effect of the injected treatments. Factors incorporated into the model as a fixed effect were ‘treatment’ (NaCl, CXB 7.7 μM, CR, CR + 7.7 μM, CR + 77 μM, CR + 770 μM), ‘tissue’ (NP and AF), and their interaction. Random effects ‘dog’ (dog 1–18) and ‘study’ (study 1 and 2) were incorporated to capture the correlation between multiple measurements within one dog. Residual plots and quantile–quantile (QQ)-plots were used to check for possible violations of normality assumptions. In case of violation, data were logarithmically transformed. The Cox proportional hazards regression model was used to estimate the effect of the injected treatments on gene expression levels. Calculations were performed on Ct values for each target gene and the mean Ct value of three reference genes was incorporated into the model as a covariate. If proportional hazard assumptions were violated, the ratio of the Ct values for each target gene to the mean Ct value of the reference genes was used for analysis. Ct values ≥ 40 were right censored. Regression coefficients were estimated by the maximum likelihood method. Model selection was based on the lowest Akaike information criterion (AIC). Confidence intervals were calculated and stated at the 99 % confidence level to correct for multiple comparisons. Differences between treatments were considered significant if the confidence interval did not include 0, whereas hazard ratios were considered significant if the confidence interval did not include 1.

## Results

### Rheological properties and handling of the pNIPAAM MgFe-LDH hydrogels

**Fig. 3:**
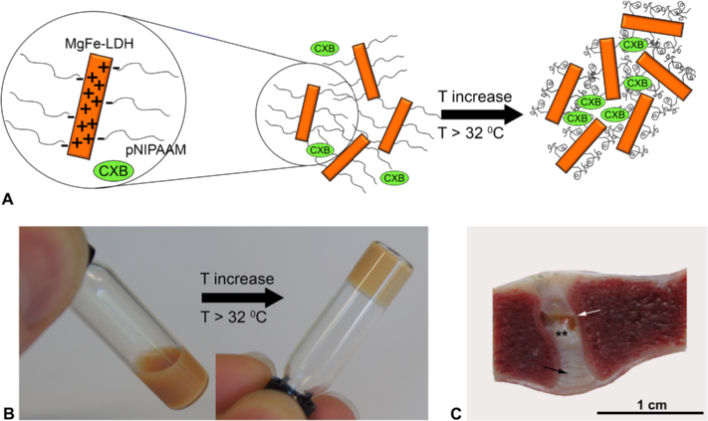
**a** Schematic diagram of the formation of stable hybrid hydrogels. Positively charged layered double hydroxides (LDH) in a pNIPAAM solution (pNIPAAM chain) transit from a low-viscous state to a stable hydrogel due to hydrophobic interactions between the isopropyl groups of pNIPAAM upon increasing temperature. The celecoxib (CXB) is present in the hydrogel in small crystals, forming a depot, from which dissolution and diffusion takes place. **b** At 37 °C gelation occurs within 10 s. **c** One month after intradiscal injection the pNIPAAM MgFe-LDH hydrogel (*white arrow*) is visible in the nucleus pulposus (NP; **). The annulus fibrosus (AF) is indicated with a *black arrow. LDH* layered double hydroxide, *pNIPAAM* poly-N-isopropylacrylamide

At low temperatures (22 °C) the complex modulus |G*| = 10 Pa and at high temperatures (37 °C) the complex modulus |G*| = 2 kPa. Typical mechanical properties of a NP are in the range of 7–21 kPa [38, 39]. However, this hydrogel was not intended to be used as a replacement for the NP and therefore no load-bearing properties were needed. The slightly lower modulus of the hydrogel was sufficient for the purpose of controlled drug release, and for the intradiscal injection of a small volume, without increasing intradiscal pressure with inherent effects on homeostasis of the resident cells. Sterilization with gamma radiation did not have a significant effect on the rheological properties of the gel (Fig. 1b). The viscous hydrogel transitioned from a low-viscous state to a stable hydrogel state within 10 s due to the hydrophobic interactions between the isopropyl groups of pNIPAAM upon increasing the temperature above its lower critical solution temperature (LCST) 32 °C (Fig. 3a and 3b). The low viscosity solution could easily be injected at room temperature via a 29G needle.

### Degradation and controlled release of CXB from pNIPAAM MgFe-LDH hydrogels in vitro

A cumulative release of 14 % CXB from hydrogels loaded with 10 mg/ml CXB was shown after 31 days, whereas a cumulative release of 11 % Mg was detected, the former indicating that CXB is still present in the hydrogel and controlled release of CXB is most probably accomplished over more than 31 days. The release of CXB showed a similar pattern as dissolution of the MgFe-LDH particles in PBS/0.2 % Tween® (Additional file 1). An increase in the concentration of Tween® is known to accelerate gel degradation and increase CXB solubility and resulted in a threefold higher Mg release and a two- to threefold higher release of CXB (Additional file 1) [22]. Neither an increase in the amount of LDH particles, nor the charge of Mg affected the CXB release profile. Furthermore, a 1.5-fold increase in the cumulative release of CXB could be detected in gels with a 6 mg/ml loading dose of CXB compared with a 10 mg/ml loading dose. However, the absolute amount of CXB was comparable (20 % of 10 mg/ml versus 30 % of 6 mg/ml) (Additional file 1). Hydrogels with higher amounts of LDH particles showed a lower amount of cumulative Mg release (Additional file 1). Degradation of pNIPAAM MgFe-LDH hydrogels and controlled release of CXB in vitro are illustrated in the figures depicted in Additional file 1.

Agarose–cell constructs incubated with 10 μM CXB bolus for 2 consecutive days apparently had taken up CXB by diffusion, leading to detectable amounts of CXB in the medium up to 7 days, which then dropped to zero (Fig. 2b). CXB released from the 0.1 mg/ml loaded hydrogels into the culture medium ranged from 1.1 to 4.2 μM (Additional file 1). CXB released from hydrogels loaded with 1 mg/ml CXB resulted in CXB concentrations in the medium ranging from 3 to 15.9 μM. To determine the activity of the released CXB, cells in the constructs were stimulated by 10 ng/ml TNF-α, which resulted in detectable PGE_2_ levels in the culture medium at day 9. Application of the 10 μM bolus of CXB during 2 consecutive days resulted in suppression of PGE_2_ levels from day 9 to 11, and PGE_2_ levels started to increase afterward to similar levels as the TNF-α-stimulated constructs. In the constructs cultured in the presence of 1 mg/ml CXB-loaded hydrogels, TNF-α-induced PGE_2_ production was completely inhibited throughout the whole culture period of 28 days (Fig. 2c).

### In vivo biocompatibility in mice

**Fig. 4:**
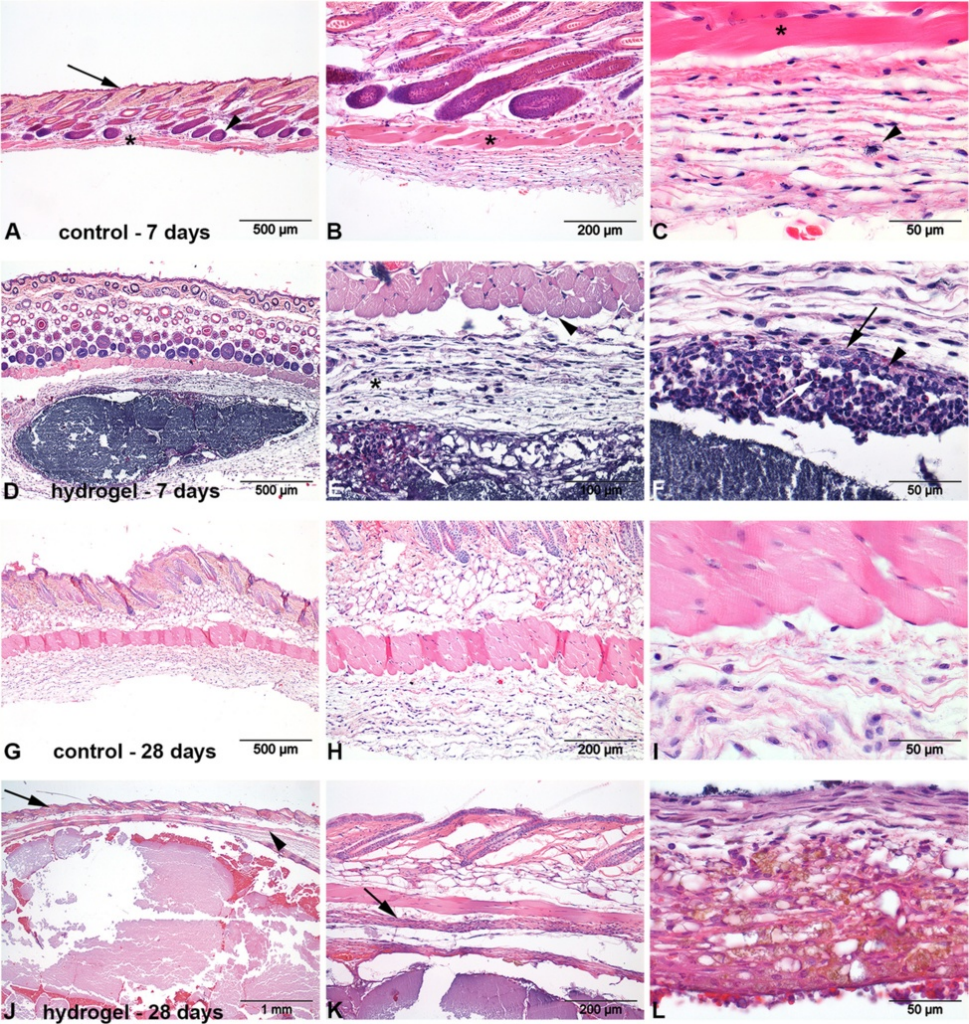
Representative histological images of subcutaneous injection sites in mice (hematoxylin and eosin stain). **a**-**c** show the skin and subcutis of a control animal at 7 days revealing the epidermis (*arrow* **a**), hair follicles (*arrowhead* **a**) and striated muscle (panniculus carnosus; *asterisk* a and b). The panniculus carnosus (*asterisk* **c**) and an occasional mastcell (*arrowhead* **c**) are visible in the subcutis. **d**-**f** show the skin 7 days after injection of the hydrogel. The hydrogel is visible as a grey, granular substance (*white arrow* **e**) positioned below the panniculus carnosus (*arrowhead* e) surrounded by a capsule of loosely arranged fibroblasts (*asterisk* **e**). Multifocally infiltrates of eosinophils (*white arrow* **f**), neutrophils (*arrowhead* **f**) and macrophages (*black arrow* **f**) separating the hydrogel from the fibrous capsule. **g**-**i** show the skin of a control animal at 28 days after injection without significant pathological changes. **j**-**l** depict the histological changes in the subcutis 28 days after injection of the hydrogel with the epidermis and panniculus carnosus indicated by an *arrow* and *arrowhead* respectively in **j**. **k** shows a more compact fibrous capsule (*arrow*) compared to the loose capsule seen after 7 days postinjection. The cellular reaction directly surrounding the hydrogel show a more granulomatous nature indicated by the presence of macrophages often containing brown pigment **l**

Subcutaneous injection of the different hydrogels showed no adverse local or systemic effects. At 7 days postinjection a fibrous capsule of varying thickness (57–76 μm) was present in all hydrogel-injected tissue samples. At the interface between the hydrogel and this capsule mainly neutrophils and macrophages were present, consistent with an acute/subacute pyogranulomatous reaction (Fig. 4). At 28 days postinjection the hydrogel and fibrous capsules were also present. In two samples a decreased thickness of the fibrotic capsules (35 and 40 μm) was observed, whereas in one sample a thickened capsule (236 μm) was observed. In all three samples macrophages constituted the predominant cell type in the intermediate layer, consistent with a granulomatous reaction. Some of these macrophages showed marked evidence of phagocytic activity. In two samples injected with PBS (control), a slight increase in macrophages was seen at day 28 compared with day 7. Giant cells, necrosis, neovascularization, and fatty infiltration were not observed at either time point.

### Intradiscal application and controlled release of CXB-loaded hydrogels in laboratory beagle dogs

#### Surgical follow-up

Before surgery a total of 162 IVDs were graded on MR images. A total of 9/162 IVDs were assigned a grade I according to the Pfirmann system, whereas 153/162 IVDs were assigned a grade II, of which 88 were injected in this study. Six out of nine dogs in the first study were ambulant the day after the injections of the test substances in the IVD and showed a slight reduction in spinal reflexes that recovered within the following 7 days. Three dogs also showed reduced weight bearing of the left hind limb, and received pain medication for a longer period of time. Two dogs that received pain medication for 7 more days, recovered completely. In one of these dogs slight dehiscence of the wound was detected and antibiotics were given for a total of 14 days. In one dog the reduction in spinal reflexes and weight bearing of the left hind limb persisted and this dog was also treated with gabapentin 5 mg/kg p.o. q.12.h. All dogs in the second study showed uneventful recovery from surgery, were ambulant the next day and showed minor reductions in spinal reflexes that recovered within 7 days.

### IVD integrity

Postmortem, Thompson score grade II was assigned to 87/88 IVDs; a grade III was assigned to 1/88 IVDs, which had been injected with the unloaded hydrogel. In 17/52 IVDs injected with the hydrogel, the tan-colored hydrogel was visible in the mid-sagittal sections of the NP (Fig. 3c). Histological evaluation of the total of 88 IVDs was performed as per the grading scheme according to Bergknut et al. [31]. Scores ranged from 4 to 14. The median histological grade in the first study was 9.5 (4–13) and in the second study 11 (8–14). No significant differences were found between the injected treatments. In one of the IVDs injected with the empty hydrogel (level L6-L7), fibrotic tissue was present in the dorsal AF; in another IVD injected with the empty hydrogel (level L7-S1), the central parts of both sides of the endplates (EPs) were very irregular and clusters of chondrocytes were present in the dorsal AF at both sides of the AF-EP interface. In one of the IVDs injected with NaCl (level T12-T13), acellular material was detected in the ventral AF. At macroscopic examination slight bulging of the ventral AF was noticed.

**Fig. 5:**
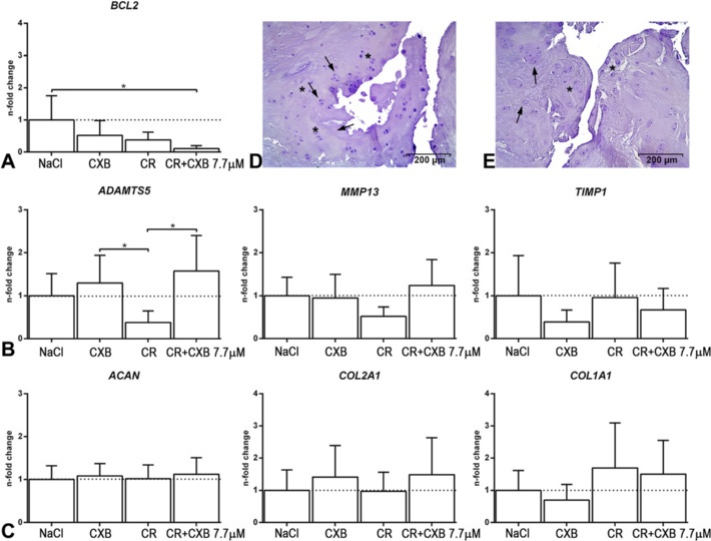
**a-c**. Relative gene expression levels per treatment. The NaCl (sham) treatment in nucleus pulposus (NP) tissue is set at 1. **a** Relative gene expression levels of *ADAMTS5* were significantly upregulated in the NP samples treated with the celecoxib (CXB) bolus (CXB 7.7 μM) and the CXB-loaded hydrogel (CR + CXB 7.7 μM) compared with the unloaded hydrogel (CR). **b** Gene expression levels *ACAN, COL2A1*, *COL1A1* did not significantly differ between treatments*.* **c** *BCL2* expression levels were significantly downregulated in the CXB-loaded hydrogel compared with the sham (NaCl). Data are expressed as n-fold changes ± standard deviation. **d** and **e** Representative histological images of early degenerated canine NP injected with NaCl (**d**) or a 770 μM celecoxib (CXB)-loaded pNIPAAM MgFe hydrogel (**e**) stained with a COX-2 antibody and counterstained with hematoxylin. No significant differences were found between the injected treatments. Chondrocyte-like cell (*asterisk*) density is increased, and small size clones are present (*arrow*), indicative of early IVD degeneration. None of the cells in these NPs demonstrated positive staining for the COX-2 antibody. Treatments: NaCl = NaCl (sham); CXB 7.7 μM = celecoxib bolus; CR = unloaded pNIPAAM MgFe-LDH hydrogel; CR + CXB 7.7 μM = pNIPAAM MgFe-LDH hydrogel loaded with 7.7 μM CXB). *Indicates significant difference at a 99 % confidence level. *ACAN* aggrecan, *ADAMTS5* a disintegrin and metalloproteinase with thrombospondin motifs 5, *BCL2* B-cell lymphoma 2, *COL1A1* collagen type 1 alpha 1, *COL2A1* collagen type 2 alpha 1, *COX* cyclooxygenase, *IVD* intervertebral disc, *pNIPAAM* poly-N-isopropylacrylamide

Relative gene expression levels of *BCL2*, a regulatory gene of cell death (apoptosis) were significantly downregulated in the CXB-loaded hydrogel compared with the sham (HR *=* 8.28, CI 99 % 1.45–47.19) (Fig. 5a). However, gene expression levels of other apoptotic markers, i.e., *CAV1*, *CASP3*, and *FasL* showed no signs of increased apoptosis in any of the treatments. Percentages of TUNEL-positive cells per total cell count showed no differences between treatments either (median 0 %, range 0–82 %). Furthermore, there were no significant differences in gene expression levels of notochordal cell markers *T*, *CK8*, *CK18*, nor in levels of *AXIN2*, *c-Myc*, and *CCND1*, associated with the Wnt pathway between the treatments.

### Extracellular matrix metabolism

**Fig. 6:**
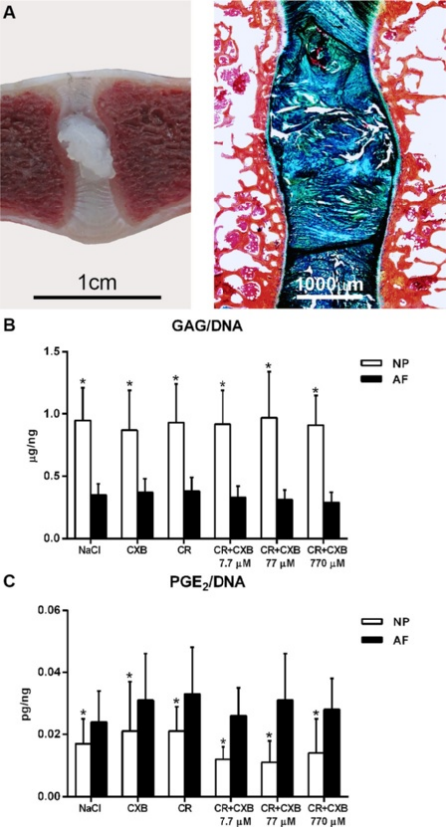
Representative macroscopic and histopathologic image stained with alcian blue/picrosirius red of an IVD treated with NaCl (**a**) and GAG and PGE_2_ levels normalized for DNA in nucleus pulposus (NP) and annulus fibrosus (AF) tissue (**b-c**). The NP in figure (**a**) has a bulging aspect due to the processing method. **b** GAG/DNA levels were significantly higher in the NP than in the AF in all treatments. **c** In all treatments PGE_2_/DNA levels were significantly lower in the NP than those in the AF. Data are expressed as mean values ± standard deviation. Treatments: NaCl = NaCl (sham); CXB 7.7 μM = celecoxib bolus; CR = unloaded pNIPAAM MgFe-LDH hydrogel; CR + CXB 7.7 μM = pNIPAAM MgFe-LDH hydrogel loaded with 7.7 μM CXB; CR + 77 μM = pNIPAAM MgFe-LDH hydrogel loaded with 77 μM CXB; CR + CXB 770 μM = pNIPAAM MgFe-LDH hydrogel loaded with 770 μM CXB). *Indicates significant difference at a 99 % confidence level. *CXB* celecoxib, *GAG* glycosaminoglycan, *IVD* intervertebral disc, *LDH* layered double hydroxide, *PGE*_*2*_ prostaglandin E_2_, *pNIPAAM* poly-N-isopropylacrylamide

Relative gene expression of the catabolic gene *ADAMTS5* was significantly upregulated in the NP samples treated with the CXB bolus (HR = 10.35, CI 99 % 1.74–61.57) and the CXB-loaded hydrogel (HR = 10.66, CI 99 % 1.70–66.67) (Fig. 5b) compared with the unloaded hydrogel. Gene expression levels of other catabolic (*MMP13*) and anti-catabolic (*TIMP1*) genes were not significantly different between treatments (Fig. 5b)*.* However, genes associated with extracellular matrix (ECM) components, i.e., *ACAN, COL2A1*, *COL1A1* did not significantly differ between treatments (Fig. 5c). These findings were consistent with normalized GAG content (GAG/DNA) in the NP as well as the AF, which did not significantly differ between treatments 4 weeks postinjection. GAG/DNA levels were significantly higher in the NP than in the AF in all treatments (M = 0.58, SD = 0.03, CI 99 % 0.50–0.66) (Fig. 6b).

### COX pathway and PGE_2_ levels

In all CXB-loaded hydrogels, a decrease in PGE_2_/DNA levels in the NP was detected relative to the NaCl-injected NPs, with a maximum reduction of 35 % for the 77 μM CXB-loaded hydrogel. However, PGE_2_/DNA levels in the NP as well as the AF showed high standard deviations and were not significantly different between the treatments 4 weeks postinjection. Gene expression levels of genes involved in PGE_2_ biosynthesis, i.e., *PTGES1*, *PTGES2*, *COX1*, and *COX2*, showed no significant differences between treatments either. Relative gene expression levels of genes associated with inflammation, i.e., *TNFA*, *IL1B*, *IL6,* and *IL10* were below the detection level for all conditions. Cells expressing positive immunohistochemical COX-2 staining were detected in only three sites out of a total of 162 (54 IVD levels), Percentages of COX-2-positive cells in the NP and VAF of two IVDs injected with 770 μM CXB-loaded hydrogel were low, 0.5 % and 2 % respectively, and 0.6 % in an IVD injected with unloaded hydrogel. Regardless of the treatment, PGE_2_/DNA levels in the NP were significantly lower than those detected in the AF (M = −0.64, SD = 0.08, CI 99 % −0.85 to −0.43) (Fig. 6c).

## Discussion

To the authors’ knowledge this is the first study that describes biocompatibility and safe intradiscal application of a thermoreversible pNIPAAM MgFe-LDH hydrogel in vivo. The hydrogel was successfully employed as a vehicle for the delivery of a COX-2 inhibitor into the IVD. The selective COX-2 inhibitor celecoxib (CXB) was selected, as this drug is commonly used to alleviate pain symptoms associated with degenerative IVD conditions.

### In vitro controlled release of CXB results into sustained suppression of PGE_2_ levels associated with inflammation

In vitro, degradation behavior of the thermoresponsive hydrogel was comparable for different loading dosages of CXB. Although an increase in the cumulative release of CXB as a percentage of the total amount loaded was detected in gels with a lower loading dose, the absolute amounts measured in the medium were comparable, demonstrating a CXB solubility-dependent release. Hydrogels with higher amounts of LDH particles showed a lower cumulative Mg release, most likely due to an increase in anionic exchange with the medium [40]. Neither an increase in the amount of LDH particles, nor changes in the charge of Mg affected the CXB release profiles. In vitro, stimulation of three-dimensional chondrocyte constructs with 10 ng/ml TNF-α, resulted in increased PGE_2_ levels in the culture medium. Furthermore, in this in vitro model, we confirmed the controlled release of CXB into the medium from 0.1 mg/ml and 1 mg/ml CXB-loaded hydrogels, respectively. Unfortunately, CXB values measured by the UPLC method showed high variances most probably due to fact that the values were measured in the lower region of the UPLC detection method [23]. TNF-α-induced PGE_2_ production was completely inhibited by 1 mg/ml CXB-loaded pNIPAAM MgFe-LDH hydrogels, thereby proving the efficacy of the CXB controlled release system to suppress in vitro PGE_2_ production for a prolonged period in contrast to the short-lived effect of a bolus of CXB.

### Safe subcutaneous and intradiscal application of the thermoreversible pNIPAAM MgFe-LDH hydrogel

In vivo, subcutaneous injection of the hydrogel did not result in local or systemic adverse effects; histology showed a moderately irritant reaction with a shift from a pyogranulomatous reaction into a granulomatous reaction with formation of a fibrous capsule, consistent with a foreign body reaction to biomaterials [41]. Based on these clinical and histological findings, in combination with the avascular nature of the IVD [42, 43], we concluded that the hydrogel would be well tolerated when applied intradiscally. Indeed, safe intradiscal injection of thermoresponsive CXB-loaded and unloaded pNIPAAM MgFe-LDH hydrogels was demonstrated in a large animal model, i.e., chondrodystrophic dogs, with naturally occurring IVD degeneration. This was corroborated by IVD histology, further determination of the anabolic/catabolic state of the ECM and cell viability by means of gene expression and biochemical analyses. The presence of the hydrogel in the NP, in contrast to the subcutaneous location, was not accompanied by a foreign body reaction. Histological findings in the majority of the canine IVDs were unremarkable. Notably, in one IVD injected with unloaded hydrogel, fibrous tissue in the AF was detected, whereas in another one, irregular EPs and clustering of chondrocytes in the dorsal AF were present. In one of the IVDs injected with NaCl, acellular material and bulging of the ventral AF were noted. These irregularities cannot be attributed with certainty to the injections [18], and may also reflect spontaneous progression of IVD degeneration. This study was conducted in mildly degenerated IVDs without fissures in the AF and we demonstrated safe intradiscal injection of 30 μl hydrogel through a 29G needle. However, we did not study the ability of the pNIPAAM MgFe-LDH hydrogel to form an interface with NP tissue and therefore we cannot exclude the risk of extrusion of the biomaterial in severely degenerated IVDs that contain annular fissures.

Viability of the resident NP cells of the NP was not affected by the in vivo intradiscal application of the pNIPAAM MgFe-LDH hydrogel loaded with CXB over a wide dose range. Relative gene expression levels of the anti-apoptotic gene *BCL2* were significantly downregulated in the NPs of IVDs injected with the CXB-loaded hydrogel compared with the NaCl-injected IVDs. In numerous cancer cell lines overexpressing COX-2, CXB has been shown to activate the intrinsic apoptotic pathway [44, 45]. However, in our study, gene expression levels of other apoptotic markers, i.e., *CAV1*, *CASP3*, and *FasL*, together with results of the TUNEL assay showed no evidence that apoptosis was affected by the intradiscal application of (un)loaded pNIPAAM MgFe-LDH hydrogel in vivo. Altogether this indicates that the differences of *BCL2* on gene expression level may not be of biological relevance. In line with the aforementioned, expression levels for notochordal cell markers *T*, *CK8* and *CK18* did not differ between groups indicating that neither the injection nor the (un)loaded biomaterial had an adverse effect on IVD health.

### Safe intradiscal application of the thermoreversible pNIPAAM MgFe-LDH hydrogel loaded with a wide range of CXB dosages

The pNIPAAM MgFe-LDH hydrogel loaded with CXB over a wide dose range also appeared to be biocompatible and safe for intradiscal application at a biomolecular and biochemical level. Intradiscal injection was not associated with IVD herniation, or with progression of degeneration. Overall, there were no significant differences between treatments on expression levels of other catabolic (*MMP13*) and anti-catabolic (*TIMP1*) genes, genes of extracellular matrix (ECM) components, i.e., *ACAN, COL2A1, COL1A1*, and the GAG/DNA content. Furthermore, gene expression levels of *ADAMTS5* were significantly upregulated in NPs treated with the CXB bolus and the CXB-loaded hydrogel, independent of the dose, compared with NPs treated with the unloaded hydrogel, suggestive of a catabolic effect of CXB. In line with this, *ADAMTS5* gene expression levels did not significantly differ between IVDs injected with unloaded hydrogels and NaCl. Our findings do not correspond with upregulated levels of *ADAMTS4* reported in bovine NPCs cultured in hyaluronan-based (HA)-pNIPAAM hydrogels compared with NPCs cultured in alginate beads. Nevertheless, these results should be compared to our results with care, as the relevant fold-change in gene expression was limited to a twofold difference in our study, and the experimental environment (i.e., in vitro versus in vivo), as well as the tissue and the composition of the hydrogel differed [46]. Altogether, the pNIPAAM MgFe-LDH hydrogel is biocompatible and can be safely injected in the IVD without affecting the IVD health based on the overall results of histological scores, immunohistochemical indices, gene expression profiles, and biochemical analyses.

### Controlled release of CXB in mildly degenerated IVDs had a limited effect on PGE_2_ levels

Over the period of 28 days and there was no dose-dependent effect in a wide range of CXB concentrations. Due to technical limitations we were not able to determine the CXB tissue levels in vivo and hence cannot elaborate on the in vivo release profile of the loaded hydrogels specifically in the matrix-rich NP environment. As CXB has a relatively high protein-binding capacity, the protein-rich NP might have enhanced CXB solubility, hence an increased release of CXB from the hydrogel [47]. Given that the only available reports on the effect of CXB on PGE_2_ production by IVD cells are from in vitro experiments, the discussion of the results is further limited by differences between cell responses in an in vivo and an in vitro situation. Furthermore, PGE_2_ levels detected in these in vitro experiments are expressed in PGE_2_/ml medium or PGE_2_/total protein, and in none of them in PGE_2_/DNA [6, 7, 9, 10, 48]. When correcting the PGE_2_ levels for protein content in the NP (Additional file 4), the results are consistent with findings in 7-day cultures of human grade 3 degenerated tissue, which showed no inhibition of PGE_2_ production in the presence of 1 μM CXB [48]. Although we cannot rule out a suboptimal effect of the CXB-loaded hydrogels in the intradiscal environment, the lack of PGE_2_ inhibition by CXB in the current study may have been attributable to the constitutive activity of COX-1 and the absence of inflammation-induced COX-2 activity. The low percentage of COX-2-positive cells detected at the immunohistochemical level supports this theory. Furthermore, in patients with degenerative joint cartilage, treatment with CXB for a period of 28 days has been shown to have a beneficial effect on GAG turnover, mainly due to COX-2 inhibition. GAG/DNA was not significantly different for any of the treatments, which may also be associated with the absence of COX-2 activity in this model. Insufficient statistical power because of the modest sample size (N = 18) may have played a role in limiting the significance of the statistical comparisons conducted (Additional file 3).

Although MR images, macroscopic, and histologic findings were consistent with mild degeneration, this phase in the degenerative cascade may actually not be associated with increased PGE_2_ levels, suggesting that inflammation occurs at a later time point, i.e., when disc protrusion and/or clinical signs are present. Incorporation of general COX inhibitors into the hydrogel may provide clarity on this.

Interestingly, regardless of the treatment condition, PGE_2_/DNA levels measured in the AF in our study were significantly higher than the levels detected in the NP for all conditions, while GAG/DNA levels in the AF were significantly lower than levels in the NP, in accordance with previous studies [5, 20]. In contrast, Miyamoto et al. described similar PGE_2_/ml levels in control NP and AF cell cultures, but showed a higher production of PGE_2_ by AF cells compared with NP cells in response to a cyclic mechanical load [49].

Inflammation is thought to play an important role in the process of IVD degeneration and disease [25]. Addressing the origin of inflammatory responses and hence an earlier stage of the degenerative cascade, may be more successful in suppressing inflammation and restoring IVD homeostasis. Emerging treatment strategies aiming at controlled release of anti-inflammatory medication that target proinflammatory mediators, e.g., TNF-α and PGE_2_ levels in the IVD, will need to focus on IVDs in human and/or veterinary patients with clinical signs of IVD disease [16, 25, 50]. PGE_2_ levels elevated in the course of a true inflammatory response in NP and/or AF tissue may be effectively decreased by selective COX-2 inhibitors, such as CXB. However, CXB release and bioavailability in the intradiscal environment should be understood in more detail. If these issues are solved, pNIPAAM MgFe-LDH hydrogels loaded with CXB might be a promising long-term treatment in patients with herniated discs and chronic low back pain that can be minimally invasive injected into the diseased IVD assisted by fluoroscopy or computer tomography.

## Conclusions

We have demonstrated the biocompatibility and safety of a pNIPAAM MgFe-LDH hydrogel via subcutaneous application in mice and via intradiscal administration in a large animal model. Furthermore, we have shown its capability for controlled delivery of the anti-inflammatory drug CXB, resulting in suppressed PGE_2_ levels in a TNF-α-stimulated three-dimensional tissue-engineered model system for up to 28 days. The controlled release of CXB from this hydrogel resulted in limited inhibition of PGE_2_ production in a large animal model with spontaneous IVD degeneration. This may be due to the stage of degeneration rather than the efficacy of the controlled release system.

## Additional files

Additional file 1:
**Synthesis and degradation of poly-N-isopropylacrylamide (pNIPAAM) MgFe-LDH hydrogels and controlled release of CXB in vitro.** (DOCX 89 kb) Additional file 2:
**Primers used for qPCR.** (DOCX 19 kb) Additional file 3:
**Power analysis of the studies in laboratory beagle dogs.** (DOCX 16 kb) Additional file 4:
**PGE**_**2**_ **normalized for total protein.** (TIFF 15443 kb)

## Abbreviations

*ACAN*: aggrecan
*ADAMTS5*: a disintegrin and metalloproteinase with thrombospondin motifs 5
AF: annulus fibrosus
AIC: Akaike information criterion
*BCL-2*: B-cell lymphoma 2
BSA: bovine serum albumin
c.r.i.: continuous rate infusion
*CASP3*: caspase 3
*CAV1*: caveolin-1
*CCND1*: cyclin-D1
*CK*: cytokeratin
*COL1A1*: collagen type 1 alpha 1
*COL2A1*: collagen type 2 alpha 1
COX: cyclooxygenase
Ct: cycle threshold
CXB: celecoxib
DEC: Animal Experiments Committee (Dutch: Dierexperimentencommissie)
ECM: extracellular matrix
*FasL*: fas ligand
FSE: fast spin echo
G*: complex shear modulus
G’: storage modulus
G”: loss modulus
GAG: glycosaminoglycan
*GAPDH*: glyceraldehyde-3-phosphate dehydrogenase
hgDMEM: high-glucose Dulbecco’s modified Eagle’s medium
i.m.: intramuscular
i.p.: intraperitoneal
i.v.: intravenous
IL: interleukin
IVD: intervertebral disc
LCST: lower critical solution temperature
LDH: layered double hydroxide
*MMP13*: matrix metalloproteinase 13
MR: magnetic resonance
NP: nucleus pulposus
p.o.: per os
PBS: phosphate-buffered saline
PGE_2_: prostaglandin E_2_
PGH_2_: prostaglandin H_2_
pNIPAAM: poly-N-isopropylacrylamide
*PTGES*: prostaglandin E synthase
q: quaque
qPCR: quantitative polymerase chain reaction
*RPS19*: ribosomal protein S19
*T*: brachyury
TBP: TATA-binding protein
TBS: Tris-buffered saline
TBST: Tris-buffered saline with 1 % Tween 20
TE: echo time
*TIMP1*: tissue inhibitor of metalloproteinase 1
TNF-α: tumor necrosis factor alpha
TR: repetition time
TUNEL: terminal deoxynucleotidyl transferase dUTP nick-end labeling
UPLC: ultra-performance liquid chromatography
