# Supplementary material for: Biocompatibility and intradiscal application of a thermoreversible celecoxib-loaded poly-N-isopropylacrylamide MgFe-layered double hydroxide hydrogel in a canine model
Source: Arthritis Res Ther. 2015 Aug 20;17(1):214. doi: 10.1186/s13075-015-0727-x (PMC4545995; doi:10.1186/s13075-015-0727-x)
Supplement: Additional file 1: — Synthesis and degradation of poly-N-isopropylacrylamide (pNIPAAM) MgFe-LDH hydrogels and controlled release of CXB in vitro. (DOCX 89 kb) [file 13075_2015_727_MOESM1_ESM.docx]

**Additional file 1.**

**Synthesis and degradation of poly-(N-isopropylacrylamide) (pNIPAAM)
MgFe-LDH hydrogels and controlled release of CXB *in vitro***

***Synthesis of poly-(N-isopropylacrylamide) (pNIPAAM) MgFe-LDH hydrogels***

The poly(N-isopropylacrylamide) (pNIPAAM) polymer with sulfonate endgroup was synthesized through radical polymerization of NIPAAM (Sigma Aldrich, St. Louis, USA) with an initiator, 2,2’-azobis(2-methylpropionitrile) (AIBN, Fluka), and in the presence of a chain transfer reagent (CTA), HS(CH_2_)_3_SO_3_Na (Sigma-Aldrich), at a molar ratio NIPAAM/AIBN/CTA of 80/0.20/1. Methanol (120 ml) (ABS, Sigma-Aldrich) was added to 33.9 g of NIPAAM and 0.668 g CTA agent under nitrogen, and the mixture was stirred for 30 minutes. After addition of 123 mg of AIBN, the mixture was immediately brought to refluxing conditions. After a reaction time of 48 hours, the reaction mixture was cooled to room temperature and the solvent was removed by evaporation. The crude polymer was dissolved in 150 ml dioxane (Sigma-Aldrich) and precipitated by the drop wise addition to a fifteen-fold volume of diisopropyl ether (Sigma-Aldrich). This precipitation was repeated two times to make sure that NIPAAM monomers were removed. The precipitated polymer was filtered, washed two times with 500 ml of diethyl ether (AR, ≥99.5%), stabilized with butylhydroxytoluene (Biosolve BV, Valkenswaard, The Netherlands), and dried overnight at room temperature. This precipitation was repeated two times to make sure that NIPAAM monomers were removed (Yield 98%). The polymer was characterized by ^1^H NMR (^1^H NMR (CDCl_3_, 400 MHz): δ 1.0-1.2 (br, 6H), 1.3-1.9 (br, 2H), 2.1-2.2 (br, 1H), 3.9-4.1 (br, 1H), 6.0-6.6 (br, 1H)), as well as Gel Permeation Chromatography (GPC) using a GPC KD-804 column (Shodex, 300 x 8.0 mm) and polyethylene glycol calibration samples. DMF with 10 mM LiBr was used as mobile phase at a rate of 1.0 ml/min at 50°C. The GPC was equipped with a refractive index detector to determine Mn (6600 g/mol), Mw (26500 g/mol), and PDI 4.0. S-elemental analysis gave 0.65 wt% sulfur (0.70 wt% calculated).

The inorganic MgFe-LDH was synthesized by co-precipitation of the metal salts with NaOH under nitrogen atmosphere. To obtain pristine MgFe-LDH, MgCl_2_·6 H_2_O and FeCl_3_·6 H_2_O (Sigma-Aldrich) were dissolved in deionized water to a concentration of 1.05M and 0.35M, respectively, and another solution was prepared using NaOH and NaCO_3_ (Sigma-Aldrich) in deionized water to a concentration of 2.35M and 0.22M respectively. Both solutions were simultaneously added to a stirred beaker using syringe pumps, stirred for another 10 minutes and then dialyzed to remove excess salts (conductivity 108 µS/cm, pH 9,5). The purified MgFe-LDH suspension was aged at 110°C for 18 hours. The dispersions were then cooled and stored at room temperature. To obtain a 6 wt% LDH dispersion, dispersions were concentrated using a rotary evaporator.

Powder X-ray diffraction (pXRD) was used to study the structural properties of LDHs. PXRD spectra/patterns were obtained using an X-ray diffractometer (Philips X’Pert SR5068; PANalytical, Almelo, the Netherlands) with Cu K*α* radiation (λ=1.54 Å) at 45 kV and 40 mA, and at a scanning rate of 0.8°/min. Peaks were observed at the following positions: *2θ* = 11.3°(003), 22.9°(006), 34.7°(009), 37.9°, 46.8°, 59.4°(Fe), 60.8°(110).The full width at half-maximum (FWHM) of the first order peak (11.3°) was 0.36°, leading to an average size of 25 nm using the Scherrer equation. The ratio Mg:Fe was determined to be 2.6:1 using a Prodigy High Dispersion Inductively Coupled Plasma Optical Emission Spectometry (ICP-OES) system (Leeman, Illinois, USA). Standards were prepared by using multi-element (23 elements in diluted nitric acid) standard solution IV (1000 mg/l) (Merck, Darmstadt, Germany). A volume of 0.5 ml of the solutions from the degradation experiment was diluted in 100 ml aqueous 1N HNO_3_, and subsequently diluted again tenfold in 1N HNO_3_.

***In vitro* release of CXB and Mg from pNIPAAM MgFe-LDH hydrogels**

**


Figure 1.** The cumulative release (%) of celecoxib (CXB) and Mg ions from the pNIPAAM MgFe-LDH hydrogels in PBS/0.2% Tween**®**.



**Figure 2.** The cumulative release (%) of celecoxib (CXB) (**A**) and Mg (**B**) from pNIPAAM MgFe*-*LDH hydrogels with 1% wt CXB or 0.6% wt CXB, with single LDH or double LDH content, and with Mg_2.5_Fe LDH, or Mg_3_Fe LDH in PBS with 0.2% Tween 80^®^ and once in 2%. Data are expressed as mean ± standard deviation. **


Figure 3.** Celecoxib (CXB) concentrations (μM) measured in medium samples of 3D chondrocyte constructs after administering a 1 μM bolus injection of CXB (CXB bolus) for 2 consecutive days and pNIPAAM MgFe-LDH hydrogels loaded with 0.1 mg/ml CXB (CR CXB). Data are expressed as mean mean ± standard deviation.

***Heating rate employed in rheological analysis of the pNIPAAM MgFe-LDH hydrogels***

It is expected that after injection in the body, the hydrogel is heating up fast due to the high conductivity of the hydrogel. An estimate calculation (assuming the hydrogel has similar properties as water) gives the following:

Energy needed to heat up 100 μl of hydrogel = 4.185 kJ/kgK * 0.1 g * 17 K = 7.1 Joule.

Heat transfer Q= k * A * dT * d = 0.6 W/mK (thermal cond. Water) * 10^-4^ m^2^ (minimum surface of gel particle of 100 μl) * 5K (minimum temperature difference between LCST and body temperature)/2.88 * 10^-3^ m (max. radius of gel particle of 100 μl) = 0.11 J/s as the minimum rate of heat transfer.

To transfer 7,1 Joule max. 65 seconds are needed. Therefore, a heating rate of 15 degrees in 60 seconds appeared to be a good estimation for the real time situation. In the beginning, heating up will be faster due to a higher temperature gradient, and the fact that the hydrogel is in a needle, increasing the surface and decreasing the transfer radius. Altogether this is difficult to translate one-on-one to the rheological measurement.
