# Supplementary material for: Biocompatibility and intradiscal application of a thermoreversible celecoxib-loaded poly-N-isopropylacrylamide MgFe-layered double hydroxide hydrogel in a canine model
Source: Arthritis Res Ther. 2015 Aug 20;17(1):214. doi: 10.1186/s13075-015-0727-x (PMC4545995; doi:10.1186/s13075-015-0727-x)
Supplement: Additional file 2: — Primers used for qPCR. (DOCX 19 kb) [file 13075_2015_727_MOESM2_ESM.docx]

**Additional file 2. Primers used for quantitative PCR**

| **Protein** | **Forward sequence 5’ -> 3’** | **EXON** | **Reverse sequence 5’ -> 3’** | **EXON** | **Amplicon size** | **Annealing temp (°C)** | **Accession no.** |
| --- | --- | --- | --- | --- | --- | --- | --- |
| **Reference genes** |  |  |  |  |  |  |  |
| GAPDH | TGTCCCCACCCCCAATGTATC | 7 | CTCCGATGCCTGCTTCACTACCTT | 8 | 100 | 58 | NM_001003142 |
| RPS19 | CCTTCCTCAAAAAGTCTGGG | 2/3 | GTTCTCATCGTAGGGAGCAAG | 3 | 95 | 61 | XM_005616513 |
| TBP | CTATTTCTTGGTGTGCATGAGG | 1 | CCTCGGCATTCAGTCTTTTC | 1 | 96 | 61 | XM_849432.3 |
| **Target genes** |  |  |  |  |  |  |  |
| ACAN | GGACACTCCTTGCAATTTGAG | 13/14 | GTCATTCCACTCTCCCTTCTC | 14 | 111 | 62 | XM_005618252 |
| COL2A1 | GCAGCAAGAGCAAGGAC | 52 | TTCTGAGAGCCCTCGGT | 53 | 151 | 62 | XM_005636674 |
| COL1A1 | GTGTGTACAGAACGGCCTCA | 2 | TCGCAAATCACGTCATCG | 2 | 109 | 61 | NM_001003090 |
| ADAMTS5 | CTACTGCACAGGGAAGAG | 5 | GAACCCATTCCACAAATGTC | 6 | 149 | 61 | XM_846025.3 |
| MMP13 | CTGAGGAAGACTTCCAGCTT | 1 | TTGGACCACTTGAGAGTTCG | 2 | 250 | 65 | XM_536598 |
| TIMP1 | GGCGTTATGAGATCAAGATGAC | 2 | ACCTGTGCAAGTATCCGC | 3 | 120 | 66 | NM_001003182 |
| TNFA | CCCCGGGCTCCAGAAGGTG | 1 | GCAGCAGGCAGAAGAGTGTGGTG | 1 | 83 | 65 | NM_001003244 |
| IL1B | TGCTGCCAAGACCTGAACCAC | 4 | TCCAAAGCTACAATGACTGACACG | 4 | 115 | 68 | NM_001037971 |
| IL6 | GAGCCCACCAGGAACGAAAGAGA | 1 | CCGGGGTAGGGAAAGCAGTAGC | 2 | 123 | 65 | NM_001003301 |
| IL10 | CCCGGGCTGAGAACCACGAC | 3 | AAATGCGCTCTTCACCTGCTCCAC | 4 | 157 | 63 | NM_001003077 |
| PTGES1 | CCAGTATTGCCGGAGTGACCAG | 3 | AAACGAAGCCCAGGAACAGGA | 4 | 97 | 68 | NM_001122854 |
| PTGES2 | GCTCTCAAGACCTACCTGG | 3 | AGTCACTTCCTTTCCCTGG | 4 | 98 | 61 | NM_001131050 |
| COX1 | GCCCTACATGTCCTTCCAGG | 9 | TCCAAGGCATCAATGTCTCCA | 10 | 86 | 65 | NM_001003023 |
| COX2 | TTCCAGACGAGCAGGCTAAT | 7 | GCAGCTCTGGGTCAAACTTC | 8 | 112 | 60 | NM_001003354 |
| T | AGACAGCCAGCAATCTG | 5 | TGGAGGGAAGTGAGAGG | 6 | 115 | 56.5 | NM_001003092 |
| CK8 | CCTTAGGCGGGTCTCTCGTA | 9 | GGGAAGCTGGTGTCTGAGTC | 9 | 149 | 63 | XM_543639 |
| CK18 | GGACAGCTCTGACTCCAGGT | 6 | AGCTTGGAGAACAGCCTGAG | 6 | 97 | 60 | XM_534794 |
| AXIN2 | GGACAAATGCGTGGATACCT | 1 | TGCTTGGAGACAATGCTGTT | 1 | 141 | 60 | XM_548025 |
| c-Myc | GCCGGCGCCCAGCGAGGATA | 1 | GCGACTGCGACGTAGGAGGGCGAGC | 1 | 108 | 61 | NM_001003246 |
| CCND1 | GCCTCGAAGATGAAGGAGAC | 1 | CAGTTTGTTCACCAGGAGCA | 1 | 151 | 60 | NM_001005757 |
| CAV1 | CGCACACCAAGGAAATCG | 1 | AAATCAATCTTGACCACGTCG | 2 | 72 | 60 | NM_001003296 |
| CASP3 | ATCACTGAAGATGGATGGGTTGGGTT | 8 | TGAAAGGAGCATGTTCTGAAGTAGCACT | 8 | 139 | 58 | NM_001003042 |
| FasL | GGGGTCAGTCCTGCAACAACAA | 4 | ATCTTCCCCTCCATCAGCATCAG | 4 | 93 | 54 | NM_001287153 |
| Bcl-2 | TGGAGAGVGTCAACCGGGAGATGT | 3 | AGGTGTGCAGATGCCGGTTCAGGT | 3 | 87 | 62 | NM_001002949 |

Primers used for qPCR analysis of target genes *aggrecan (ACAN), collagen type II (COL2A1), collagen type I (COL1A1)*, *a disintegrin and metalloproteinase with thrombospondin motifs (ADAMTS5), matrix metalloproteinase 13 (MMP13), tissue inhibitor of metalloproteinase 1 (TIMP1*), *tumor necrosis factor-alpha* *(TNFA), interleukin-1β* *(IL1B), interleukin-6 (IL6), interleukin-10 (IL10)*, *prostaglandin E synthase 1* (*PTGES1)*, *prostaglandin E synthase 2* *(PTGES2)*, *cyclooxygenase 1* (*COX1*), and *cyclooxygenase 2 (COX2), brachyury (T), cytokeratin-8 (CK8), cytokeratin-18 (CK18),* *axin-2 (AXIN2), c-Myc (c-Myc)*, *cyclin-D1 (CCND1)*, *caveolin-1 (CAV1), caspase 3 (CASP3), fas ligand (FasL), Bcl-2 (BCL2)* and reference genes *glyceraldehyde 3-phosphate dehydrogenase* (*GAPDH*), *ribosomal protein S19* (*RPS19*), and *TATA-binding protein* (*TBP).*
