# Supplementary figures and images for: Biocompatibility and intradiscal application of a thermoreversible celecoxib-loaded poly-N-isopropylacrylamide MgFe-layered double hydroxide hydrogel in a canine model
Source: Arthritis Res Ther. 2015 Aug 20;17(1):214. doi: 10.1186/s13075-015-0727-x (PMC4545995; doi:10.1186/s13075-015-0727-x)

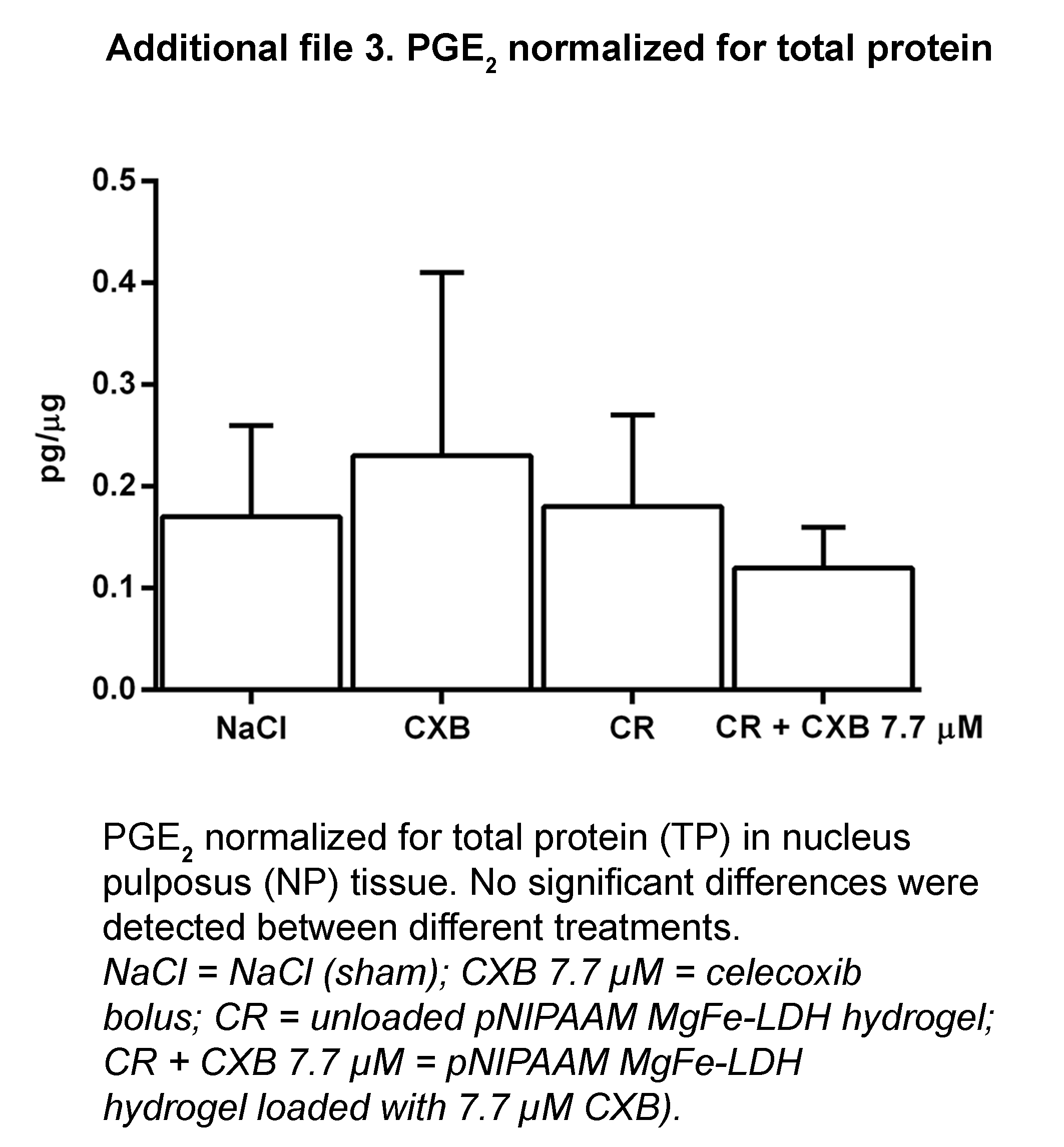

Supplement: Additional file 3: — Power analysis of the studies in laboratory beagle dogs. (DOCX 16 kb) [file 13075_2015_727_MOESM3_ESM.docx]

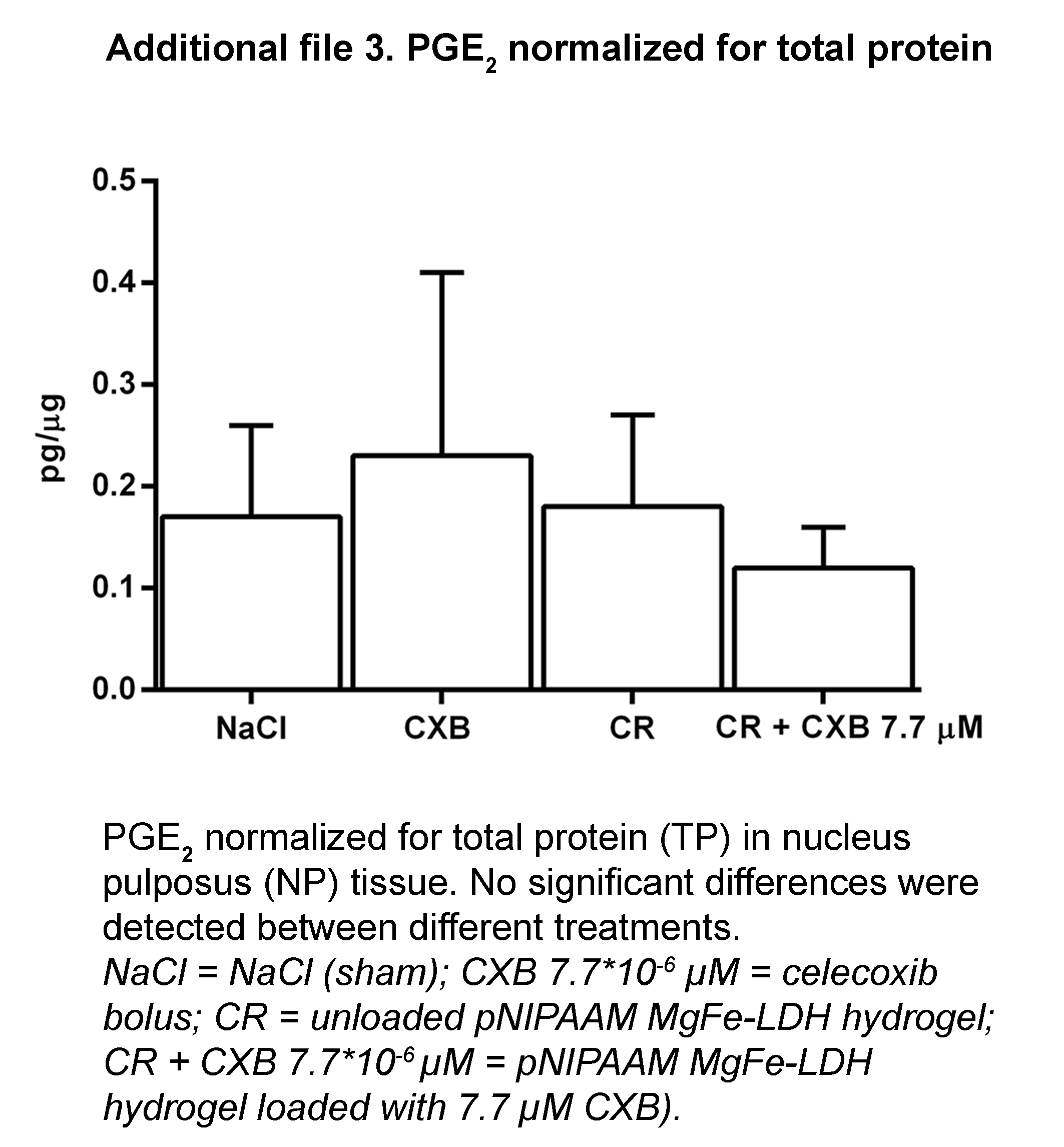

Supplement: Additional file 4: — PGE 2 normalized for total protein. (TIFF 15443 kb) [file 13075_2015_727_MOESM4_ESM.tiff]
